# Vitamin D Receptor, Retinoid X Receptor, Ki-67, Survivin, and Ezrin Expression in Canine Osteosarcoma

**DOI:** 10.1155/2012/761034

**Published:** 2012-12-30

**Authors:** John Davies, Heather Heeb, Rama Garimella, Kimberly Templeton, David Pinson, Ossama Tawfik

**Affiliations:** ^1^BluePearl Veterinary Partners, Overland Park, KS 66210, USA; ^2^Department of Veterinary Clinical Sciences, College of Veterinary Medicine, Washington State University, Pullman, WA 99164, USA; ^3^Oncology Division, Department of Medicine, Kansas University Medical Center, Kansas City, KS 66160, USA; ^4^Department of Dietetics and Nutrition, Kansas University Medical Center, Kansas City, KS 66160, USA; ^5^Department of Orthopedic Surgery, Kansas University Medical Center, Kansas City, KS 66160, USA; ^6^Department of Pathology and Laboratory Medicine, Kansas University Medical Center, 3901 Rainbow Boulevard, Kansas City, KS 66160, USA; ^7^LACF at University of Illinois College of Medicine at Peoria, One Illini Drive, Peoria, IL 61656, USA

## Abstract

Canine osteosarcoma (OS) is an aggressive malignant bone tumor. Prognosis is primarily determined by clinical parameters. Vitamin D has been postulated as a novel therapeutic option for many malignancies. Upon activation, vitamin D receptors (VDRs) combine with retinoid receptor (RXR) forming a heterodimer initiating a cascade of events. Vitamin D's antineoplastic activity and its mechanism of action in OS remain to be clearly established. Expression of VDR, RXR, Ki-67, survivin, and ezrin was studied in 33 archived, canine OS specimens. VDR, RXR, survivin, and ezrin were expressed in the majority of cases. There was no statistically significant difference in VDR expression in relationship with tumor grade, type, or locations or animal breed, age, and/or sex. No significant association (*p* = 0.316) between tumor grade and Ki-67 expression was found; in particular, no difference in Ki-67 expression between grades 2 and 3 OSs was found, while a negative correlation was noted between Ki-67 and VDR expression (*ρ* = −0.466), a positive correlation between survivin and RXR expression was found (*p* = 0.374). A significant relationship exists between VDR and RXR expression in OSs and proliferative/apoptosis markers. These results establish a foundation for elucidating mechanisms by which vitamin D induces antineoplastic activity in OS.

## 1. Introduction

Osteosarcoma (OS) is the most common primary bone tumor in dogs accounting for up to 85% of all primary malignant bone tumors [1, 2]. Canine OS is a locally aggressive neoplasm and metastasis is extremely common [1]. Metastases are typically hematogenous in route and arise early in the course of disease. While less than 15% of dogs have radiographic evidence of metastasis at the time of diagnosis and regional lymph node involvement is rare, approximately 90% of dogs diagnosed with OS would die of metastatic disease if amputation with clean margins was the only treatment [1]. The current standard of care consists of surgical excision with wide margins followed by chemotherapy for control of microscopic metastatic disease [1]. Radiation therapy has not been shown to be effective as a curative modality of treatment. It has primarily been used for palliative pain relief [3, 4]. Some have shown its value in downstaging a primary tumor prior to surgical excision [3].

Currently the most effective option for controlling metastatic disease is multi-agent chemotherapy with doxorubicin, cisplatin, or carboplatin [1, 5]. While all have demonstrated some effectiveness at controlling the growth of OS, multiple side effects such as bone marrow suppression, hepatotoxicity, nephrotoxicity, cardiotoxicity, ototoxicity, neuropathies, and anaphylaxis have been reported. Further, curative rates are low with only 20% of patients being successfully brought into remission [1].

Despite continued attempts to improve therapeutic success, reported median survival times have remained static at 235–540 days [1, 4] with an average median survival time of less than one year [1]. This highlights the ineffectiveness of current therapeutic regimens in treating systemic metastatic disease.

An effective approach to treating neoplastic diseases such as OS is the induction of differentiation through the use of cellular differentiation agents in neoadjuvant therapy. When compared to healthy bone cells, OS cells have a degree of anaplasia loss of the mature cell phenotype. The loss of growth control accompanying the loss of the mature cell phenotype results in uncontrolled proliferation. It has been postulated that if these anaplastic cells could be induced to differentiate into a mature phenotype, uncontrolled proliferation would be kept in check [6, 7]. Naturally occurring cases of OS spontaneously regressing [8] and in vitro studies that correlate an increase in apoptosis and reduction in proliferation with increased phenotypical differentiation [6, 9, 10] support this theory.

While the mechanisms of its action are currently incompletely understood, vitamin D has been shown to be capable of inducing differentiation and reducing proliferation in multiple neoplasms [11]. Human tumors including colon, breast, and skin cancers [6, 9, 10] and canine OS [10] have been shown to respond to vitamin D therapy. The current study is clinically significant because previous studies have validated the use of canine OS as an appropriate model for human OS [12, 13].

Dietary vitamin D is hydroxylated to its metabolically active form 1,25-dihydroxy vitamin D_3_ (1,25(OH)_2_D_3_) through a process of hydroxylation that occurs in the liver and kidneys [14]. 1,25(OH)_2_D_3_ is traditionally best known for its role in calcium and phosphorous homeostasis where it promotes absorption of these minerals from the intestinal mucosa [14]. However, recent research provided strong evidence that vitamin D has numerous effects beyond calcium and phosphorous metabolism [15], including observed anti-neoplastic effects [6, 9–11].

Actions of 1,25(OH)_2_D_3_ are mediated by the vitamin D receptor (VDR), a nuclear phosphoprotein which binds 1,25(OH)_2_D_3_ with high affinity. This alters the ligand binding domain of the VDR and results in a strong heterodimerization with retinoid X receptors (RXR). The resulting 1,25(OH)_2_D_3_-VDR-RXR complex promotes vitamin D responsive element binding and transcription of vitamin D promoted gene products [16].

While in vitro studies have shown vitamin D induces differentiation and apoptosis in a single canine OS cell line [9, 10], the potential use of vitamin D as a treatment for canine OS is dependent on the expression of its receptors [11]. To the authors' knowledge, the expression of VDRs in spontaneous canine OS has not been previously reported.

The purpose of this study is to evaluate the expression of VDRs in archived spontaneous canine OS by immunohistochemistry. We hypothesized that VDRs would be expressed in a sufficiently large percentage of spontaneous canine OSs to support the development of vitamin D therapy as a viable adjunctive treatment option for canine OS. In addition, we attempted to correlate VDR expression with that of RXR and markers for tumor proliferation (Ki-67), apoptosis (survivin), progression, and metastasis (ezrin).

## 2. Materials and Methods

Thirty-three cases of confirmed canine OS submitted to the Veterinary Lab Resources of Kansas City Between January 2006 and February 2009 were included in this study. All samples were fixed in formalin, decalcified where necessary, and embedded in paraffin wax. Paraffin blocks are sectioned at 4 microns mounted on Superfrost + slides and baked in a 65°C oven for one hour. After deparaffinization, VDR, RXR, Ki-67, survivin, and ezrin slides were heat-treated in citrate buffer pH 6.0 for five minutes using the Biocare Decloaking Chamber (pressure cooker). After pressure returned to zero, slide jars were removed from the Chamber, cooled in the buffer for 10 minutes, and then transferred to tris-buffered saline with Tween. All staining was performed at room temperature using a Dako Autostainer per company protocol. Endogenous peroxidase was blocked using 3% hydrogen peroxide for 10 min then rinsed. Primary antibodies' vendor, titer, incubation time and detection methods are noted in Table 1. Antibodies were diluted using Dako Antibody Diluent. All slides were counterstained with Mayer's Hematoxylin, dehydrated, cleared, and coverslipped with permanent mounting media. MIB-1 is a monoclonal Ki-67 antibody. Ki-67 is a nuclear protein only expressed in cycling cells and is a sensitive marker of cellular proliferation [17]. RXR is a nuclear protein that binds various retinoids (a family of polyisoprenoid lipids which includes vitamin A) [18]. It is an intrinsic component of the VDR complex required for transcription of vitamin D responsive gene products. Ezrin is a cytoskeletal cross linking protein involved in cell adhesion, cell survival, and cell motility and has been linked to metastasis [19]. Survivin is a member of the inhibitor of apoptosis protein family and is not present in most normal adult differentiated tissues. Survivin is expressed during the G2/M phase of the cell cycle and inhibits apoptosis [20].

Representative sections from each tumor were stained with hematoxylin & eosin (H&E) and the above mentioned panel of immunohistochemical stains. Appropriate positive and negative controls for each marker were included. Positive controls for the markers were selected from surgical specimens received in the surgical pathology laboratory and their positivity was confirmed when compared with other samples.

**Figure 1:**
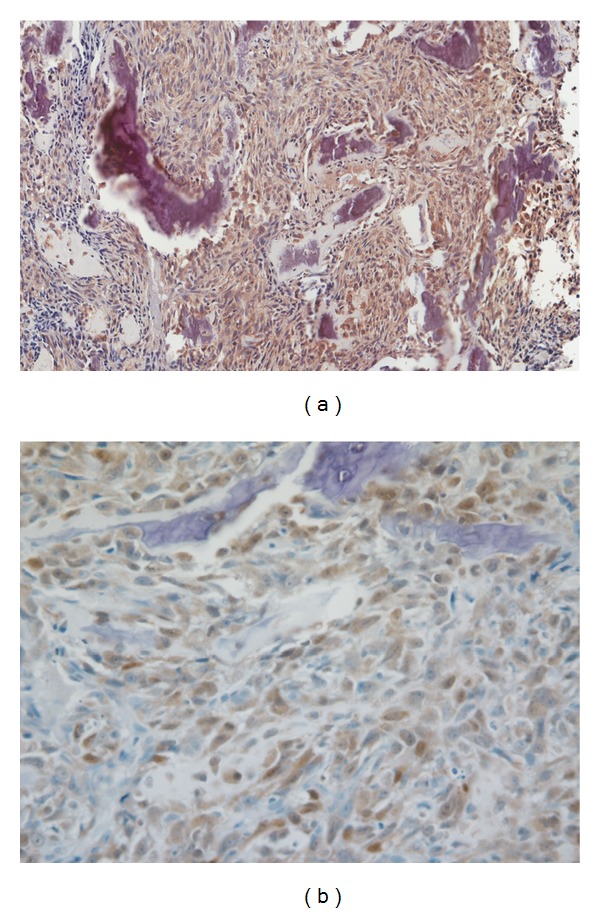
Representative photomicrograph of VDR expression in canine osteosarcoma (immunostain 20x (a) and 40x magnification (b)).

**Figure 2:**
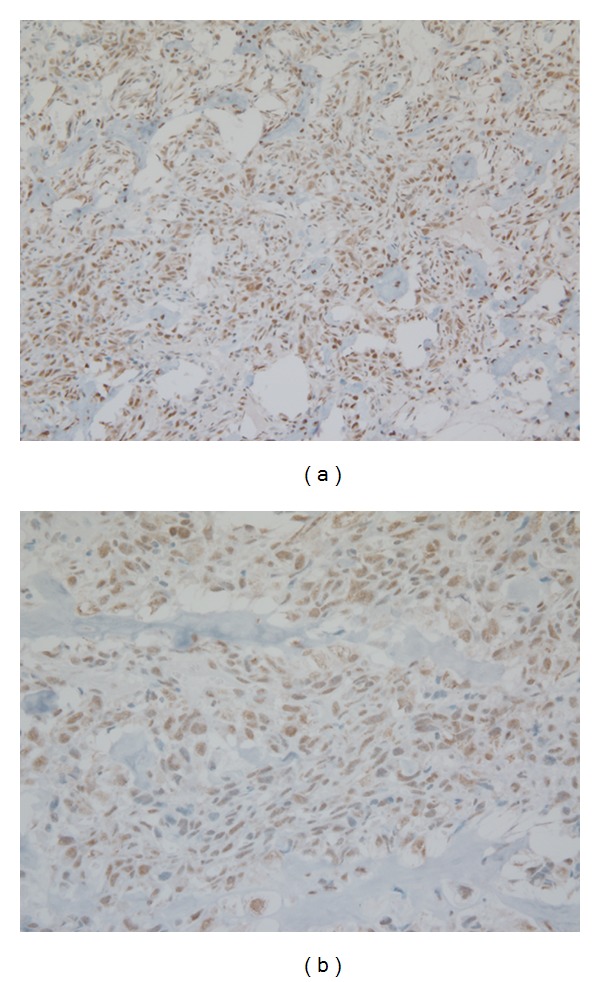
Representative photomicrograph of RXR expression in canine osteosarcoma (immunostain 20x (a) and 40x magnification (b)).

**Figure 3:**
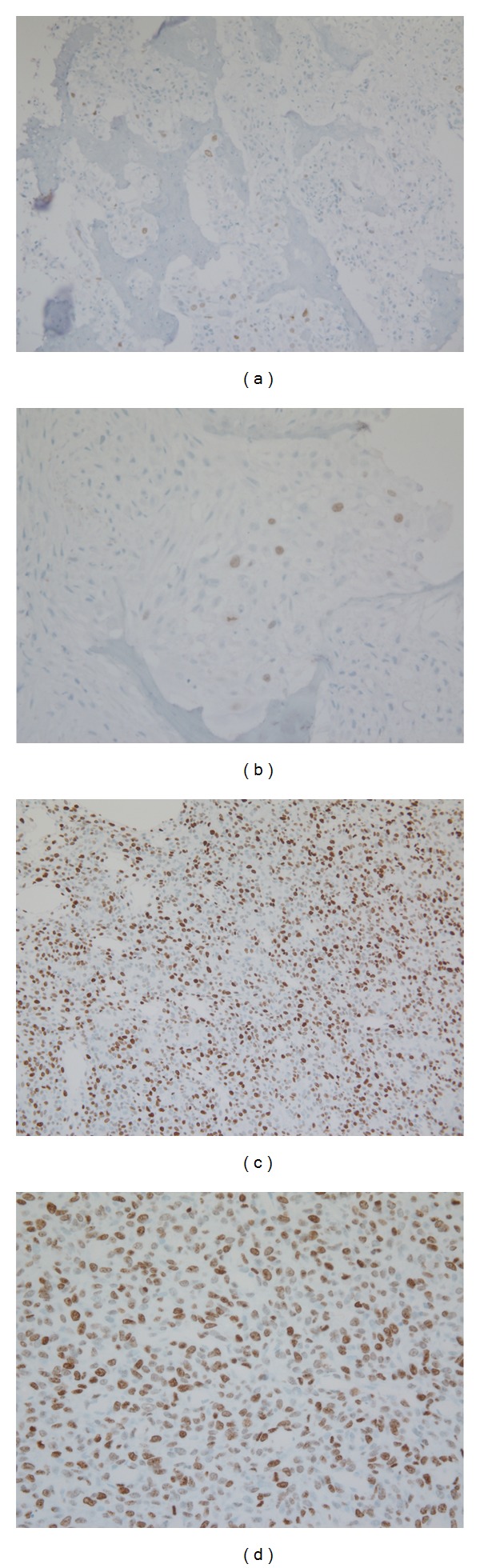
Representative photomicrographs of Ki-67 expression in canine osteosarcoma (immunostain 20x and 40x magnification). Low expression in (a) and (b) and high expression in (c) and (d).

**Figure 4:**
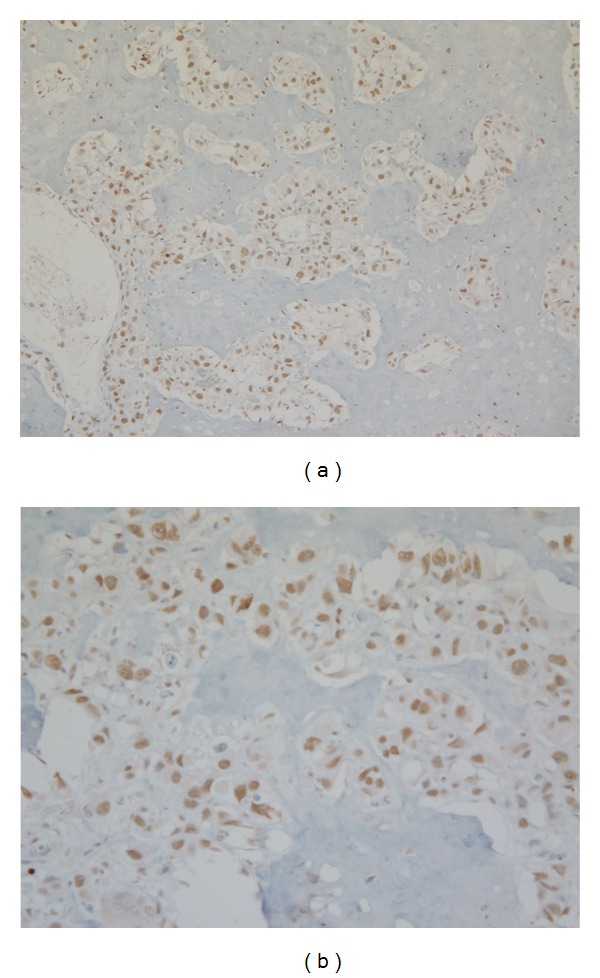
Representative photomicrograph of survivin expression in canine osteosarcoma (immunostain 20x (a) and 40x magnification (b)).

**Figure 5:**
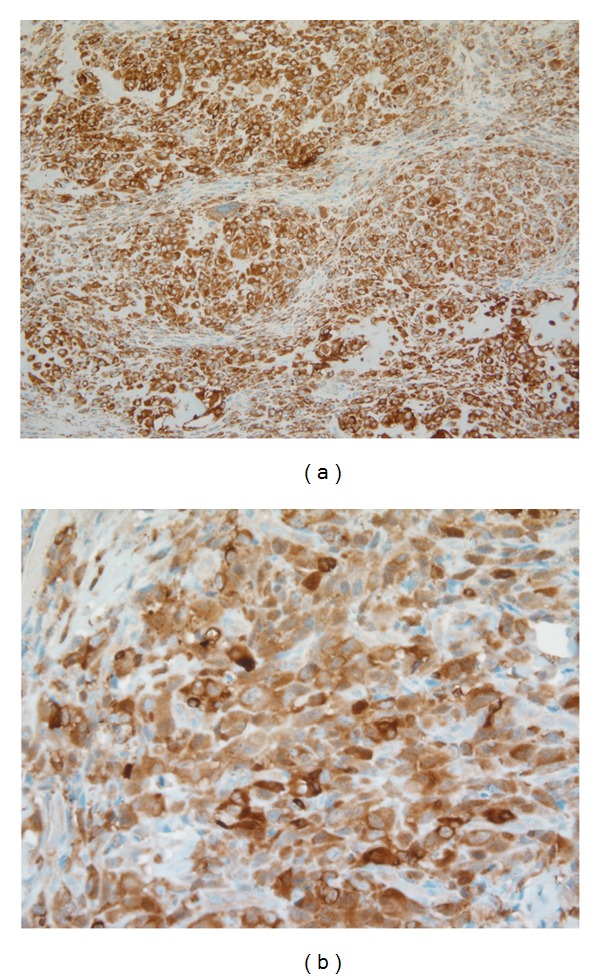
Representative photomicrograph of ezrin expression in canine osteosarcoma (immunostain 20x (a) and 40x magnification (b)).

All slides were read simultaneously by two of the authors (OT and JD). The original diagnosis of OS was confirmed and a nuclear and histological grade (1–3) was assigned to each sample based on the degree of differentiation, production of osteoid, mitotic index, cellular and nuclear pleomorphism, necrosis, and invasiveness observed. Positive immunohistochemical reactions are defined as a positive cytoplasmic staining for VDR and ezrin (Figures 1 and 5, resp.) and positive nuclear staining for RXR (Figure 2), Ki-67 (Figure 3), and survivin (Figure 4). The method of Grizzle et al. was used to determine the immunostain score for VDR and ezrin [21]. Cytoplasmic staining intensity was scored from 0 to 3. The percent of positive cells at each intensity level was multiplied by the appropriate intensity score; these values were summed and then divided by the total number of cells to obtain a weighted average score between 0 and 3. A score of 0 indicated “negative” results, and 1+, 2+, or 3+ represented “positive” results. This method has been proven to be more representative than the usual 0–3+ staining intensity as it takes into consideration both antigen presence and changes in antigen expression for the lesion. It also takes into account all relevant areas on the slides. For proliferation index (PI) of Ki-67 and percent positivity for RXR and survivin, the percentage of nuclei with immunopositivity was determined using the PI program of the ChromaVision Automated Cellular Imaging System (ACIS) (San Juan Capistrano, CA). A staining of 10% or more for cells staining with Ki-67 was considered positive, whereas for RXR and survivin, any counts greater than or equal to 5% were considered positive.

Statistical analysis was conducted using a combination of the Mann-Whitney *U* test and a two tailed Spearman's rank correlation test [22]. The Mann-Whitney *U* test was used to determine if the difference between 2 data sets, for example, expression of VDR between grades 2 and 3 OSs, (*p*  values ≤ 0.05), was considered significant. The Spearman's rank correlation test was used to determine if there was a significant correlation between two continuous factors such as VDR expression and MIB-1 expression. Alpha < 0.05 was considered significant which corresponded to *ρ* < −0.346, >0.346 representing a significant correlation. Data was analyzed using Minitab v. 15 (Minitab Inc., State College, PA, USA).

## 3. Results

The median age of the dogs was 10 years (mean 9.3 years, Range 5–15 years). Breeds included were Labrador retriever (10), Greyhound (5), Rottweiler (5), Mixed breed (4), Great Pyrenees (2) Golden retriever (2), German shepherd (2), American pit bull (1), Collie (1). Tumor locations were humerus (9), radius/ulna (4), scapula/shoulder (4), Mandible (3), maxilla (4), femur (1), ilium (1), tarsal bones (1), tibia (4) and unknown location (1).

All tumors were either grade II (5) or grade III (28). Four were chondroblastic, four were fibroblastic, one contained both chondroblastic and fibroblastic cell populations, and one was a giant cell OS. Nuclear grade was identical to the histologic grade in 32/33 samples and not significantly different (*p* = 1.0). As a result, only the histological grade was further evaluated in this study.

VDR was expressed in 76% (25/33) of the tumors analyzed in this study (Table 2). No statistically significant difference in VDR expression was found between histological tumor grades (*p* = 0.23), nor was a significant association between breed (*p* = 0.097), age (*p* = 0.073), sex (*p* = 0.068), or tumor location (*p* = 0.  015) found. Though not statistically significant (*p* = 0.058), 3 of 4 chondroblastic osteosarcomas expressed a high level of VDR (2+) compared to osteoblastic tumors (4/23).

No significant association (*p* = 0.316) between tumor grade and Ki-67 expression was found, nor was there a significant difference in Ki-67 expression between grades 2 and 3 OSs (*p* = 0.791). In addition, no correlation between Ki-67 and tumor location was noted (*p* = 0.147). Interestingly, the tumors with the highest Ki-67 expression (≥25%) were grade III OSs and did not express VDR and when analyzed, a significant negative correlation was found between Ki-67 expression and VDR expression (*ρ* = −0.466).

RXR was well expressed (≥25%) in 97% (31/32) of the tumors (Table 2). The exceptions occurred in rare samples and were attributed to decalcification artifact. While low (≤4%) levels of survivin were consistent with the observation of decalcification artifact, no correlation was found between survivin expression and tumor grade (*p* = 0.076), VDR expression (*p* = 0.330), or Ki-67 (*p* = 0.104). Interestingly, a positive correlation between survivin and RXR expression was found (*p* = 0.374). Ezrin was uniformly expressed well (≥2+) with the exception of a single sample which was attributed to the decalcification process.

## 4. Discussion

Differentiation therapy as a method of controlling neoplastic growth is a concept that has recently generated much interest. Vitamin D in particular has been the subject of intensive research, both in its mechanism of action and its effects on various human neoplasms [6, 7, 11, 18, 23–25]. Multiple studies have demonstrated that vitamin D can affect the rate of proliferation, degree of apoptosis, and the phenotype of numerous neoplastic cell lines. These studies report variable effects and degrees of response between different tumor types and within cell lines of the same tumor type [6, 9–11]. In a study by Valrance et al. the proapoptotic and antiproliferative effects of vitamin D were induced in some, but not all breast cancer cell lines. Valrance et al. noted that the nonresponsive cell lines were those that lacked expression of VDR [26]. While the mechanisms by which vitamin D induces apoptosis and inhibits proliferation in neoplastic cell lines not clear, the success of vitamin D therapy is dependent to a large extent on the expression of the VDR within the target population of cells [7, 11, 26].

Barroga et al. reported the stimulation of a canine OS cell line with vitamin D analogs and retinoids to differentiate into a more mature phenotype that showed evidence of increased apoptosis and decreased cell proliferation [9, 10]. While their results support the efficacy of vitamin D as a therapeutic agent for canine OS, all studies were conducted on cell lines derived from a single spontaneous canine OS. Since the success of vitamin D therapy depends on the expression of the VDR, it is critical to determine whether VDR is expressed in spontaneous OSs to warrant further research into vitamin D as a potential therapeutic agent. To the authors' knowledge, the expression of VDR in spontaneous canine OSs has not been previously reported.

Our results show that VDR was expressed in 73% (25/33) of spontaneous canine OSs with 27% (9/33) showing 2+ VDR expression. These results are also in agreement with our recently reported immunohistochemical data confirming that VDRs are expressed in 86.4% of primary and metastatic human OS tumors (95 of 110 tumors) [27]. While the level of VDR expression necessary for a clinical response is not known, these results suggest that vitamin D therapy may be effective in the majority of naturally occurring canine and human OSs. Additionally, the high levels of VDR expressed by chondroblastic OSs were very interesting. While this was not statistically different from the other OS subtypes (*p* = 0.056) this may be due to sample size limitations. Further studies with larger sample sizes may show a significant difference indicating an increased susceptibility of chondroblastic OSs to vitamin D therapy.

It has been shown that VDR expression can be upregulated. Krishnan and Feldman showed that VDR expression in a breast cancer cell line was upregulated by stimulation with a combination of serum and growth factors [28]. If VDR expression can similarly be induced in canine OS, the number of cases susceptible to vitamin D therapy may be significantly increased.

A study by Cross et al. [29] studying human colonic neoplasia found that VDR expression increased in parallel with dedifferentiation in the early phases of carcinogenesis whereas only low levels of VDR were expressed in late stage carcinomas. Cross et al. suggested that colon cancer cells were responding to the stimulation of tumor cell proliferation by increasing production of anti-proliferative substances, such as those mediated through VDRs. The low levels of VDR in late stage carcinomas were attributed to failure of the antiproliferative effect of vitamin D and subsequent loss of VDR expression. High levels of VDR in colorectal cancer have also been associated with improved survival [24].

No significant difference (*P* = 0.23) was found between VDR expression and tumor grade in the current study. These findings were similar to our previously reported results in human OS [27]. Interestingly, a negative correlation between VDR expression and Ki-67 (*P* = −0.466) was noted, supporting the theory that more aggressive OSs express less VDR. Expanding this study to include a larger sample size and OSs of all grades is necessary to determine if there is a link between tumor grade and VDR expression in canine OS.

RXR is an integral component of the RXR-VDR-Vitamin D complex necessary to promote expression of vitamin D responsive gene products. If insufficient RXR is present, it is possible that the tumor may be nonresponsive to vitamin D therapy as a result of limiting RXR levels [23]. In this study, as well as in our human OS study [27], RXR was consistently well expressed (≥25% in 30/32 cases) and found in all cases that stained positive for VDR with the exception of 3 cases, where the lack of expression was attributed to decalcification artifact. It should be pointed out however, that high levels of RXR were also present in samples not expressing VDR. While our sample size is limited, this study does not support RXR expression as a limiting factor in the response of canine OS to vitamin D therapy.

All samples were additionally stained for the cytoskeletal protein ezrin. Ezrin has been linked to metastasis in human breast carcinoma, human OS, and a mouse model of OS [19, 30–32]. Further, high ezrin expression in canine and human OS has been reported to be associated with the early development of metastasis [19, 32]. Consistent with previous reports [19], ezrin was strongly expressed in all samples (≥2+) with the exception of a single case in which poor staining was attributed to decalcification artifact. These findings correlate well with the known behavior of canine OS to metastasize early [1]. Unfortunately, data was not available in this study to correlate ezrin levels with the presence of metastases. Ezrin is interesting, however, as it could potentially be used as a prognostic indicator and as a measure of treatment success. Low levels of ezrin would be expected to correlate with lower grade tumors that are less likely to metastasize. With successful differentiation therapy, metastases should be delayed/prevented and decreased ezrin levels could be observed. Further research would be needed to determine if ezrin staining could be used in this manner.

Canine OS is currently histologically graded in part by assessing the number of mitotic figures per 10 high power fields [2]. While this an objective measure of cells undergoing mitosis, it does not allow recognition of cycling cells outside the mitotic phase. MIB-1 is a Ki-67 immunostain that stains all actively growing cells [17], allowing for rapid, objective, and sensitive assessment of actively cycling cells within the tumor. As high Ki-67 levels correlate with a rapidly proliferating OS, decreased survival times would be expected. While, survival data was not available for the cases in our study, a study by Jong et al, did not find a correlation between Ki-67 expression and survival in human OS [33]. Ki-67 does not appear to be useful for predicting survival; however, it would be useful as a sensitive quantitative marker of cellular proliferation.

Survivin is a nuclear protein and a member of the inhibitors of apoptosis family. High levels of survivin are found in tumors with unregulated growth and low levels of apoptosis and have been correlated with decreased survival in human OS patients [20, 34]. High survivin levels were expected in this study as the majority of samples were high grade OSs. Survivin was well expressed (>25%) in 21/31 samples. While there was a wide range in expression (0–98%) of survivin, our study did not find a correlation between survivin expression and tumor grade (*ρ* = −0.07).

As survival data was not available in this study, we were unable to evaluate a correlation between survivin expression and survival. Evaluating survivin levels should be included in future studies assessing effects of vitamin D therapy, as its levels would be expected to decrease significantly in response to vitamin D's proapoptotic effects.

Interestingly, a weak positive correlation between survivin and RXR expression was found (*p* = 0.374). As there was not a significant difference in survivin (*p* = 0.20) or RXR (*p* = 0.82) expression between histological grades, this weak correlation was attributed to the fact that both RXR and survivin were both consistently well expressed in all samples.

Vitamin D has great potential for the treatment of canine OS. As a differentiation inducing agent, its effects would be systemic and address both local and metastatic disease. Further, when compared to the cytotoxic effects of chemotherapy, vitamin D's side effects are theoretically less severe and reversible, relating primarily to a dose dependent induced hypercalcemia [11, 35].

Though current research supports the antiproliferative and proapoptotic effects of vitamin D on numerous neoplasms including canine OS [6, 7, 9–11, 23, 26, 36], no clinical studies evaluating the response of dogs or humans with OS to vitamin D have been reported. There is however considerable concern that therapeutic level of vitamin D may not be attainable without the development of an unacceptable degree of hypercalcemia. With this in mind, studies are being conducted with the aim of developing calcemic vitamin D analogs [18, 36]. Working from a different angle, others are investigating synergistic compounds such as retinoids aimed at potentiating the antiproliferative and/or antineoplastic effects of vitamin D [9, 36, 37].

While the degree of VDR expression necessary for a response and the amount of vitamin D necessary to achieve a response is a subject for future research, our findings support our hypothesis that a significant number of spontaneous canine OSs (73%) express VDRs and are potential candidates for vitamin D therapy. Further, as canine OS is validated as a model for human OS [13], these findings can potentially be extrapolated to human patients.

## 5. Conclusion

In conclusion, we demonstrated the presence of VDR in the vast majority of archival paraffin-embedded canine OS samples. A significant relationship exists between VDR and RXR expression in OSs and proliferative/apoptosis markers. These results would establish a foundation for elucidating mechanisms by which vitamin D induces its antineoplastic activity. Our ongoing studies are focused on elucidation of molecular basis of vitamin D mediated prodifferentiation, proapoptotic, and antimetastatic effects of vitamin D in human OS cell lines. Results of this continued work, as well as recent research showing synergism in treating canines with OS with vitamin D and chemotherapy, may result in improved responsiveness to therapy and overall survival while decreasing treatment side effects.

## Figures and Tables

**Table 1:** Individual antibodies, vendor, titer, time of titration, epitope retrieval method, and method of detection.

Antibody	Manufacturer	Titer/Incubation (min)	Antigen retrieval Pretreatment	Detection
VDR	Santa Cruz Biotechnology, Inc, Santa Cruz, CA	1 : 500/30′′	None	Envision + anti-rabbit, Dako, Carpineria, CA
RXR Retinoid Receptor	Lab Vision Neomarkers, Fremont, CA	1 : 300/60′′	Citrate pH 6.0, Biocare Decloaking Chamber 5′′	Envision + anti-mouse, Dako, Carpinteria, CA
Ki-67 (MIB-1)	Dako, Carpinteria, CA	1 : 200/30′′	Citrate pH 6.0, Biocare Decloaking Chamber 5′′	Envision + anti-mouse, Dako, Carpinteria, CA
Survivin	Lab Vision, Fremont, CA	1 : 50/60′′	Citrate pH 6.0, Biocare Decloaking Chamber 5′′	Envision + anti-rabbit, Dako, Carpinteria, CA
Ezrin	Lab Vision, Fremont, CA	1 : 200/30′′	Citrate pH 6.0, Biocare Decloaking Chamber 5′′	Envision + anti-mouse, Dako, Carpinteria, CA

**Table 2:** Immunohistochemical expression of VDR, RXR, Ki-67, surviving, and ezrin in canine osteosarcoma specimens.

Marker	Cutpoint value used for expression	Proportion of cells expressing marker (percentage)*	Mean intensity of staining^#^ or percent positivity (range)
VDR	>0	25/33 (76%)	0.88 (0–2+)
RXR	≥5%	31/32 (97%)	76.5% (0–99%)
Ki-67	≥10%	15/26 (58%)	13.8% (1–75%)
Survivin	≥5%	25/31 (81%)	52.5% (0–98%)
Ezrin	>0	33/33 (100%)	2.7 (2-3+)

*Percentage of tumors expressing the marker within each group and number of positive samples compared to the total number of samples studied.

^#^Mean staining intensity of VDR in each group.
